# Evaluation of a Bayesian dosing calculator for vancomycin in pediatric patients with augmented renal clearance

**DOI:** 10.3389/fped.2025.1683683

**Published:** 2025-12-03

**Authors:** Abdullah Alsultan, Muneera Al-Jelaify, Huda Alshahrani, Noura M. Alajmi, Ghadeer Alfuhaydi, Saeed Alqahtani, Ali Somily, Mashal M. Almutairi, Manal Abouelkheir

**Affiliations:** 1 Department of Clinical Pharmacy, College of Pharmacy, King Saud University, Riyadh, Saudi Arabia; 2 Clinical Pharmacokinetics and Pharmacodynamics Unit, King Saud University Medical City, Riyadh, Saudi Arabia; 3 Pharmacy Services, King Saud University Medical City, Riyadh, Saudi Arabia; 4 College of Pharmacy, King Khalid University, Abha, Saudi Arabia; 5 Department of Pharmaceutical Care Services, King Abdulaziz Medical City, Jeddah, Saudi Arabia; 6 College of Pharmacy, Qassim University, Qassim, Saudi Arabia; 7 Department of Pathology, College of Medicine, King Saud University and King Saud University Medical City, Riyadh, Saudi Arabia; 8 Department of Pharmacology and Toxicology, College of Pharmacy, King Saud University, Riyadh, Saudi Arabia; 9 Department of Clinical Pharmacy, Faculty of Pharmacy, Misr International University, Cairo, Egypt

**Keywords:** pediatrics, augmented renal clearance, vancomycin, Bayesian dose calculators, model-informed precision dosing

## Abstract

**Background:**

Augmented renal clearance (ARC) is increasingly recognized among pediatric oncology and intensive care patients. It can result in subtherapeutic concentrations of renally eliminated drugs like vancomycin. Bayesian dosing tools are recommended to individualize therapy, yet their performance in pediatric ARC remains underexplored. This study evaluated the predictive accuracy of the freely available Bayesian dosing calculator, NextDose, for vancomycin in pediatric patients with and without ARC. NextDose is a web-based application built on a large-population pharmacokinetic model encompassing neonates to adults with varying renal function.

**Methods:**

A retrospective observational study included pediatric patients (1–12 years) who received vancomycin and had at least one steady-state serum concentration. ARC was defined as estimated glomerular filtration rate (eGFR) > 130 mL/min/1.73 m^2^. Predictive performance was assessed using relative median prediction error (rMPE, bias) and relative median absolute prediction error (rMAPE, precision; lower values indicate higher precision). Both *a priori* (using only clinical/demographic data) and a posteriori (drug-level informed) predictions were evaluated.

**Results:**

A total of 112 pediatric patients were included, of whom 47 (42%) met ARC criteria. The mean age was 5.9 ± 3.4 years; 10.7% were younger than 2 years, 57.2% were aged 2–7 years, and 32.1% were 7–12 years old. *a priori* predictions showed high bias (rMPE 27%) and moderate precision (rMAPE 31%), with no significant differences between the ARC and non-ARC groups. In contrast, a posteriori predictions demonstrated marked improvement (rMPE −3.9%, rMAPE 13.5%), with 86% of predictions meeting the <30% prediction-error threshold. Patients with ARC exhibited superior predictive accuracy than non-ARC counterparts (rMAPE 12% vs. 17.5%, *p* = 0.03).

**Conclusion:**

NextDose overestimated vancomycin concentrations in *a priori* predictions, suggesting it may not be suitable for initial dose calculations. Incorporating one or two measured concentrations significantly improved predictive accuracy, particularly in patients with ARC, supporting its use alongside therapeutic drug monitoring to personalize vancomycin monitoring.

## Background

Augmented renal clearance (ARC) is well-recognized but often underappreciated in pediatric patients. ARC in pediatric patients is typically defined as an estimated glomerular filtration rate (eGFR) exceeding 130–160 mL/min/1.73 m^2^, most commonly calculated using the Schwartz formula (1–3). ARC is particularly prevalent among patients in pediatric intensive care units (PICUs) and pediatric oncology settings (4, 5). Studies have reported that ARC may affect up to 8%–78% in PICU patients (5). ARC results from hemodynamic alterations, including increased cardiac output and renal blood flow, as well as systemic vasodilation driven by the release of inflammatory mediators such as cytokines and prostaglandins. In addition, aggressive fluid resuscitation and the use of vasoactive agents can enhance cardiac preload, further contributing to elevated renal perfusion and augmented clearance (1). Risk factors associated with ARC in pediatric patients include low serum creatinine, febrile neutropenia, male sex, older pediatric age (e.g., adolescents), septic shock, and the use of vasoactive medications (5).

In patients with ARC, standard dosing regimens of renally eliminated drugs often result in subtherapeutic concentrations, increasing the risk of treatment failure and the emergence of antimicrobial resistance (1, 5–8). Despite this clinical concern, most dosing guidelines focus on patients with normal or impaired renal function, providing limited guidance for those with ARC. Patients with ARC have high eGFR and might require larger doses of renally eliminated drugs (1, 7, 9).

One such drug is vancomycin, a glycopeptide antibiotic widely used to treat serious Gram-positive infections in the pediatric population. These infections are associated with high mortality and morbidity, particularly in vulnerable populations such as those in the PICU and oncology wards. The drug has high inter- and intra-patient variability and a narrow therapeutic index. Dosing is especially challenging in patients with ARC, with multiple studies identifying it as a risk factor contributing to subtherapeutic vancomycin concentrations (8, 10). For example, a previous study has reported vancomycin trough concentrations below 5 mg/L in 35.2% of ARC cases (4). These studies highlight the importance of individualized dosing and close monitoring of vancomycin.

One approach to improve the dosing and therapeutic drug monitoring (TDM) of vancomycin is to use model-informed precision dosing (MIPD) or Bayesian dose calculators. These tools have emerged as a promising strategy to personalize antimicrobial therapy in pediatric patients. They are increasingly used to tailor dosing based on individual pharmacokinetic profiles, potentially improving target attainment (11–16). Historically, vancomycin dosing was guided by trough concentrations; however, recent consensus guidelines recommend targeting an area under the concentration-time curve over the minimum inhibitory concentration (AUC₂₄/MIC) of 400–600 to optimize efficacy and minimize nephrotoxicity (17). Bayesian forecasting facilitates estimation of AUC values using one or two measured concentrations, allowing precise, patient-specific dosing adjustments without extensive sampling. The 2020 IDSA/ASHP/SIPD/PID vancomycin dosing and monitoring guidelines specifically recommend the use of Bayesian software to achieve these AUC-based targets (17). However, the predictive performance of these calculators in pediatric patients with ARC remains largely unknown.

Given the high prevalence and clinical implications of ARC, especially in vulnerable subpopulations such as PICU and oncology patients, it is important to evaluate whether current Bayesian dosing tools can reliably predict vancomycin concentrations in these populations. This study aims to evaluate the predictive accuracy of a freely available online Bayesian dose calculator in pediatric patients with ARC and to compare its performance to that in patients without ARC.

## Method

### Study design

This was a single-center retrospective observational study conducted at King Saud University Medical City, Riyadh, Saudi Arabia. The inclusion criteria were pediatric patients aged 1 to 12 years who received vancomycin for a suspected or confirmed Gram-positive infection with at least one measured drug concentration available at the steady state. Patients were classified as having ARC if their eGFR exceeded 130 mL/min/1.73 m^2^ (5). eGFR was calculated using the bedside Schwartz equation (18). Collected data included demographic and clinical variables such as age, weight, height, sex, hospital ward, oncology status, vancomycin indication and dosing information (dose, frequency and administration time), as well as the timing and values of serum drug concentrations. Vancomycin concentrations were determined at the pharmacokinetic laboratory within King Saud University Medical City using the Architect i4000SR immunoassay analyzer (Abbott), with a standard calibration curve ranging from 3 to 100 mg/L. As per the hospital protocol, vancomycin peaks are to be collected one hour after the end of vancomycin infusion and trough levels are to be collected 30 min prior to the next dose. Serum creatinine was also measured as part of the routine clinical practice in the hospital laboratory using the Jaffe method.

### Prediction accuracy of the Bayesian model

To assess predictive accuracy, we used the freely available online dose calculator NextDose (https://www.nextdose.org/) to predict vancomycin concentrations. Up to date, it is the only available open-access Bayesian dose calculator that incorporates a pediatric-specific vancomycin model. For each patient, the accuracy of the calculator was evaluated for both *a priori* and posteriori predictions. *a priori* is a term that refers to the use of a population pharmacokinetic model that is based solely on the patient's baseline demographic and clinical characteristics (e.g., age, weight, renal function) to calculate an initial dosing regimen. In contrast, a posteriori involves also integrating drug concentrations to refine individual PK estimates and perform dose adjustments accordingly. The vancomycin model implemented in NextDose is based on the population PK model described in a recent publication by Holford et al. (19). This joint population PK model for gentamicin, amikacin, and vancomycin was developed using the extensive “GAVamycin” dataset, which includes 9,901 patients ranging from premature neonates to adults across 18 studies. Key covariates incorporated in the model include: total bodyweight, postnatal age, postmenstrual age and GFR. These covariates influence both clearance and volume of distribution. Weight and age primarily affect size and maturation, whereas GFR is the principal physiological determinant of renal clearance. The model aims to utilize GFR as the key physiological parameter to distinguish the components of renal clearance for these agents, with the ultimate goal of supporting individualized dosing.

### Prediction-based diagnostics and statistical analysis

We evaluated prediction accuracy using established metrics of bias and precision. Bias was calculated as the relative median prediction error (rMPE), and precision was assessed using the relative median absolute prediction error (rMAPE), where lower values indicate higher precision, using the following formulas:

$$r\begin{array}{c} \mathrm{MPE}\;(\text{\%})=\frac{1}{N}\cdot \sum_{i=1}^{N}\left(\frac{C_{i\_\mathrm{Predicted}}-C_{\;i\_\mathrm{Observed}}}{C_{i\_\mathrm{Observed}}}\right)\times 100 \end{array}$$

$$\mathrm{rMAPE}\;(\text{\%})=\frac{1}{N}\cdot \sum_{i=1}^{N}\left|\frac{C_{i\_\mathrm{Predicted}}-C_{\;i\_\mathrm{Observed}\;}}{C_{\;i\_\mathrm{Observed}}}\right|\times 100$$

The criteria for acceptance were set as follows:

For both *a priori* and a posteriori predictions, bias (rMPE) was considered acceptable if <10% (20). While no universally accepted threshold exists for precision (rMAPE) has been established in the literature, values <30% are commonly regarded as indicative of acceptable predictive performance for a posteriori prediction (21, 22). We also calculated the percentage of observations that met the predefined precision criterion (<30% prediction error).

While the primary objective of the analysis was to evaluate the predictive accuracy across the entire cohort of pediatrics and to compare accuracy for ARC vs. non-ARC patients, we conducted additional analyses to explore potential sources of variability in predictive accuracy. The categorical variables that we tested included:

- Oncology vs. non-oncology patients;
- PICU vs. general ward admissions;
- Male vs. female
- Patients with one vs. two measured concentrations during the initial dosing occasion.

Continuous variables we tested included age, eGFR and body weight.

Continuous data are presented as mean ± standard deviations (SD), while categorical variables are presented as frequencies and percentages. Comparisons of predictive accuracy between categorical groups were performed using independent t-tests, while the influence of continuous variables on predictive accuracy was evaluated using simple linear regression. Categorical variables were compared using the Chi-square test. *P*-value <0.05 was considered statistically significant. All statistical analyses were conducted using the R statistical software (version 4.3.1).

## Results

### Baseline demographics

A total of 112 pediatric patients were included in the analysis. The mean age was 5.9 ± 3.4 years, with 10.7% of patients under 2 years, 57.2% between 2 and 7 years, and 32.1% between 7 and 12 years. The average weight was 19.8 ± 12.9 kg, and the mean height was 108.5 ± 24.4 cm. Males accounted for 55.4% of the cohort. The mean serum creatinine was 35.4 ± 17.8 µmol/L, and the average eGFR, calculated using the Schwartz bedside equation, was 130.2 ± 24.3 mL/min/1.73 m^2^. Oncology patients made up 15.2% of the cohort, while 42% were PICU patients. The most common indications for vancomycin therapy were sepsis or septic shock (43%), followed by pneumonia (12.5%), meningitis (11.6%), and febrile neutropenia (10.7%). The mean total daily vancomycin dose was 59.6 ± 10.5 mg/kg/day. At the first sampling occasion, a total of 185 vancomycin concentrations were measured: 73 peak levels (mean 20.4 ± 5.3 mg/L) and 112 trough levels (mean 12 ± 6.6 mg/L). On the second sampling occasion, a total of 69 observations for 53 patients were available, comprising 24 peaks and 45 trough samples.

As shown in Table 1, among the 112 patients, 47 (42%) met the ARC definition criteria. Compared to non-ARC patients, those with ARC had significantly lower serum creatinine levels (24.8 ± 7.6 vs. 43.2 ± 19.1 µmol/L, *p* < 0.01) and higher eGFR values (177.5 ± 48.1 vs. 96.1 ± 25.0 mL/min/1.73 m^2^, *p* < 0.01), consistent with enhanced renal function. Patients with ARC were also generally older (6.4 ± 3.7 vs. 5.9 ± 4.0 years) and had slightly higher body weight (21.2 ± 13.1 vs. 18.8 ± 12.7) than the non-ARC group, although these differences did not reach statistical significance (*p* > 0.05). Although oncology diagnoses were more common in the ARC group (23.4% vs. 9.2%, *p* = 0.07), this did not reach statistical significance. No major differences were observed in sex distribution, infection types, or PICU admission rates between groups. The overall total vancomycin daily dose was slightly higher in ARC patients; they had slightly lower average peak and trough levels, although not significant.

**Table 1:** Baseline demographics and clinical characteristics.

Variable	All patients (*n* = 112)	ARC (*n* = 47)	Non-ARC (*n* = 65)	*p*-value
Male sex	62 (55.4%)	27 (57.4%)	35 (53.8%)	0.85
Age (years)	5.9 ± 3.4	6.4 ± 3.7	5.4 ± 4.0	0.14
Age group:				
<2 years	12 (10.7%)	6 (12.8%)	6 (9.2%)	0.9
2–7 years	64 (57.2%)	25 (53.2%)	39 (60%)	
>7 years	36 (32.1%)	16 (34.0%)	20 (30.8%)	
Weight (kg)	19.8 ± 12.9	21.2 ± 13.1	18.8 ± 12.7	0.33
Height (cm)	108.5 ± 24.5	113.7 ± 23.7	104.7 ± 24.1	0.055
SCr (umol/L)	35.5 ± 17.9	24.8 ± 7.6	43.2 ± 19.1	<0.01
GFR (mL/min/1.73 m^2^)	130.2 ± 54.6	177.5 ± 48.1	96.1 ± 25.0	<0.01
Oncology	17 (15.2%)	11 (23.4%)	6 (9.2%)	0.07
PICU	47 (42%)	23 (48.9%)	24 (36.9%)	0.28
Indications:				0.34
Sepsis & Septic shock	48 (42.9%)	17 (36.2%)	21 (32.3%)	
Febrile neutropenia	12 (10.7%)	8 (17%)	4 (6.2%)	
Meningitis	13 (11.6%)	4 (8.5%)	9 (13.8%)	
Pneumonia	14 (12.5%)	6 (12.8%)	8 (12.3%)	
Others	35 (31.1%)	12 (25.5%)	23 (35.3%)	
Total daily dose (mg/kg/day)	59.6 ± 10.9	61.2 ± 12.1	58.5 ± 8.9	0.2
Peak level (mg/L): Observed	20.4 ± 5.3	19.5 ± 5.1	21 ± 5.4	0.3
Trough level (mg/L): Observed	12 ± 6.6	11.2 ± 7.3	12.8 ± 6	0.41

Data expressed as mean ± SD or *n* (%), *P* value <0.05 (bold) indicates statistically significant difference.

SCr, serum creatinine; GFR, glomerular filtration rate estimated using the schwartz “bedside” equation; ARC, augmented renal clearance.

## Prediction accuracy of the Bayesian model

### *A priori* predictions

**Figure 1:**
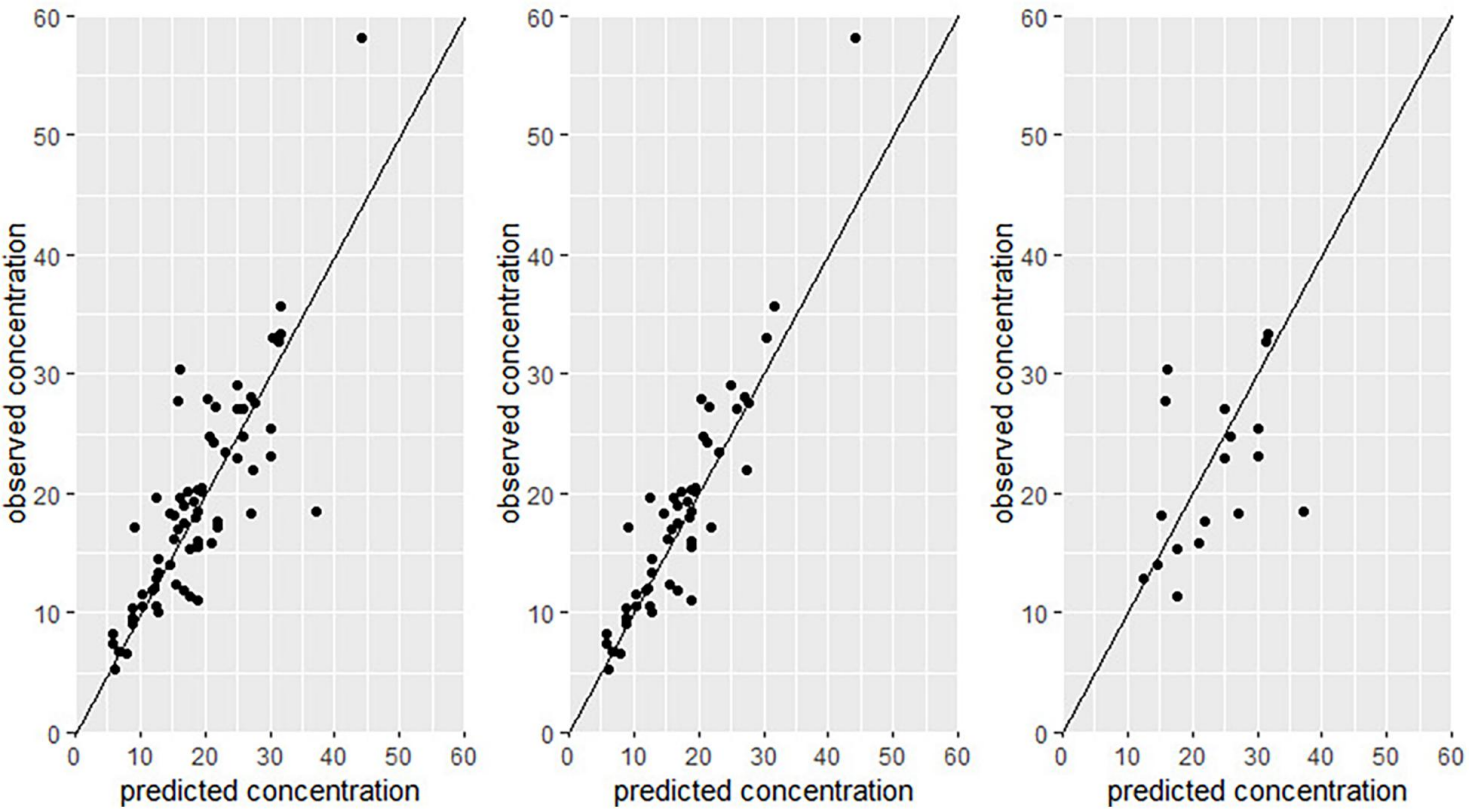
Observed vs. predicted concentration for *a priori* predictions. Left is the full cohort, Middle is the ARC and right is the non-ARC.

For *a priori* predictions (*n* = 185), the overall bias (rMPE) for the full cohort was 27% and precision (rMAPE) was 31%, with 49% of predictions achieving the predefined precision criterion of <30% prediction error. As shown in Table 2, subgroup analyses showed comparable performance between patients with and without ARC, with bias values of 25% vs. 29% in non-ARC, *p* = 0.43, and precision values of 32% vs. 31% in non-ARC, *p* = 0.65. Similarly, 47% of predictions in the ARC group and 50% in the non-ARC group achieved precision within the <30% error threshold. Figure 1 displays observed vs. predicted concentrations for the entire cohort, as well as stratified by ARC status. Oncology patients exhibited slightly higher bias (34.5%) and lower precision (37.5%) compared to non-oncology patients (bias 26.5%, rMAPE 30.5%), though these differences were not statistically significant (*p* = 0.54, *p* = 0.38). Across clinical setting, the model's predictions in PICU patients showed greater bias (30%) and lower precision (44%) relative to those in general ward patients (bias 27%, precision 29%), with only 36% of PICU predictions falling within the <30% prediction error threshold compared to 55% in the general ward group (*p* = 0.3, *p* = 0.21). Regarding the effect of sex, predictive performance was comparable between males and females, with no significant differences observed in either bias or precision. The effects of continuous variables, age and body weight, were not statistically significant (*R*^2^ = 0.014 and 0.010 for age and weight on bias, respectively; *R*^2^ = 0.012 and 0.010 for age and weight on precision, respectively.

**Table 2:** Bias and precision for *a priori* predictions.

Variable (*n* = number of subjects, number of samples)	% Bias (rMPE)	% Precision (rMAPE)	% of predictions with acceptable precision (<30% error)
Full cohort (*n* = 112, 185)	27	31	49
ARC status			
Yes (*n* = 47, 74)	25	32	47
No (*n* = 65, 111)	29	31	50
*p*-value	0.43	0.65	
Oncology status			
Yes (*n* = 16, 36)	34.5	37.5	38
No (*n* = 96, 149)	26.5	30.5	51
*p*-value	0.54	0.38	
Sex			
Male (*n*=, 103)	26.9	31	50
Female (*n*=, 82)	28.5	32	49
*p*-value	0.55	0.9	
Admission site			
PICU (*n* = 47, 55)	30	44	36
General ward (*n* = 65, 130)	27	29	55
*p*-value	0.3	0.21	

ARC, augmented renal clearance; PICU, pediatric intensive care unit.

### A posteriori predictions

In contrast, posteriori predictions (*n* = 69), which incorporate measured drug concentrations, improved due to Bayesian estimation by the model. The overall bias for the full cohort was −3.9%, and the precision was 13.5%, with 86% of predictions meeting the <30% prediction error threshold.

**Figure 2:**
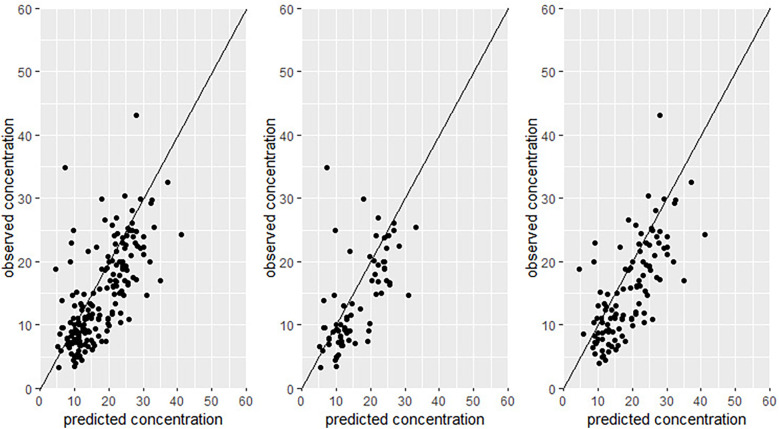
Observed VS predicted concentration for a posterioiri predictions. Left is the full cohort, Middle is the ARC and right is the non-ARC.

All subgroups showed acceptable levels of bias and precision, but there were differences in accuracy between the subgroups as shown in Table 3. ARC patients benefited significantly from having earlier measurements, with bias and precision values of −4.5% and 12%, respectively, and 92% of predictions within acceptable prediction error limits (<30% prediction error). In contrast, non-ARC patients had significantly higher bias (6.9%) and lower precision (17.5%), with only 67% of predictions achieving acceptable precision compared to ARC patients (*p* = 0.04 for bias, *p* = 0.03 for precision). Predictive performance was comparable between oncology and non-oncology patients (bias: −5.5% vs. −3%, *p* = 0.64; precision: 12% vs. 15%, *p* = 0.86). Patients in the general ward had better predictive accuracy (bias: −4.9%, precision: 9.4%) than those in the PICU (bias: 0%, precision: 17%; *p* = 0.045 for precision). Additionally, patients with two concentration samples on the first occasion had significantly higher precision (7.5%) compared to those with only one sample (17.5%), *p* = 0.02; with 97% of these predictions meeting the predefined precision criterion (<30% prediction error). Observed vs. predicted concentrations for both *a priori* and a posteriori predictions are presented in Figures 1, 2. These plots visually confirm improved alignment with tighter dispersion around the line of identity with a posteriori predictions, particularly among patients with ARC. Regarding the effects of continuous variables, age and body weight, none showed a significant correlation with bias or precision.

**Table 3:** Bias and precision for posteriori predictions.

Variable (*n* = number of subjects, number of samples	% Bias (rMPE)	% Precision (rMAPE)	% of predictions with acceptable precision (<30% error)
Full cohort (*n* = 53, 69)	−3.9	13.5	86
ARC status			
Yes (*n* = 35, 51)	−4.5	12	92
No (*n* = 18, 18)	6.9	17.5	67
*p*-value	**0**.**04**	**0**.**03**	
Oncology status			
Yes (*n* = 11, 15)	−5.5	12	87
No (*n* = 42, 54)	−3	15	85
*p*-value	0.64	0.86	
Admission site			
PICU (*n* = 38, 39)	0	17	77
General ward (*n* = 15, 30)	−4.9	9.4	97
*p*-value	0.31	**0**.**045**	
Sex			
Male (*n* = 31, 42)	−3.3	14.4	88
Female (*n* = 22, 27)	−4.4	12	81
*p*-value	**0**.**5**	**0**.**37**	
Number of samples per occasion			
One sample on the 1st occasion (*n* = 37, 37)	0	17.5	76
Two samples on 1st occasion (*n* = 16, 32)	−4.5	7.5	97
*p*-value	0.30	**0**.**02**	

Bold indicates the *p*-values for the statistical test.

ARC, augmented renal clearance; PICU, pediatric intensive care unit.

## Discussion

In this study, we evaluated the predictive performance of the freely accessible online Bayesian dose calculator (NextDose) for pediatric patients receiving vancomycin, with a particular focus on those with ARC. For *a priori* predictions (predictions based solely on demographic and clinical data), the calculator showed moderate precision but with a high level of bias, with a consistent tendency to overpredict vancomycin concentrations in all subgroups. None of the subgroups met the predefined threshold for acceptable bias, and no statistically significant differences in predictive accuracy were observed between groups at this stage. However, incorporating one or two measured concentrations markedly improved predictive performance across all subgroups, which demonstrated an acceptable level of bias (rMPE <7%) and high precision (rMAPE < 18%). These findings underscore the clinical value of Bayesian forecasting combined with therapeutic drug monitoring (TDM) to individualize dosing and suggest that, owing to the accuracy of a posteriori predictions, the need for repeated sampling may be limited.

Improved accuracy was particularly evident in specific subgroups, including patients with ARC, those managed in general wards, and those with two concentration samples obtained during the initial dosing interval. ARC patients demonstrated both lower bias and higher precision, whereas patients in the general ward and those with two samples collected during the initial dosing occasion showed significantly improved precision. The improved performance in general ward patients may be attributed to their relatively stable physiology, in contrast to the greater pharmacokinetic variability often seen in PICU patients. The improved accuracy with two samples is expected, as additional data points enhance model fitting and reduce predictive uncertainty. However, the superior accuracy observed in ARC patients is less straightforward. The model implemented in NextDose is based on a large and diverse dataset comprising 3,232 patients with 8,877 vancomycin observations across a wide age range from premature neonates to elderly adults with varying serum creatinine levels. This extensive and heterogeneous dataset may contribute to the model's robustness and its capacity to adjust for clearance variability, including the increased clearance observed in patients with ARC.

It is challenging to pinpoint the exact reason for the high bias observed in the *a priori* predictions, as none of the subgroups achieved acceptable levels of bias or precision. One possible explanation is that the underlying model was developed using a population with different demographic or clinical characteristics, suggesting potential interethnic variability. Additionally, reliance on retrospectively collected routine TDM data may have introduced variability that contributed to the observed error. Nonetheless, it is well-documented that *a priori* predictions often exhibit substantial bias, consistent with findings from previous studies. These results align with earlier research demonstrating the limitations of pharmacokinetic models when relying solely on demographic and clinical variables without incorporating drug concentration data. For instance, a study by Lv et al. evaluated the predictive performance of 10 population pharmacokinetic models in pediatric patients (23). None of the models demonstrated acceptable levels of bias or precision in the absence of drug concentration data. Predictive accuracy improved substantially when one or two observed drug concentrations were incorporated to inform subsequent predictions (a posteriori). Similarly, Frymoyer et al**.** demonstrated that Bayesian forecasting significantly improves target attainment for vancomycin in children, particularly when one or more drug levels are used for dose adjustment. This highlights the value of a posteriori predictions (24). On the other hand, Le J. et al. raised concerns that not all Bayesian tools are equally effective across different age groups and that their predictive performance may vary depending on the underlying population PK models they use (25). Zhao et al. found that while Bayesian models improve dose individualization, they may still underperform in neonates or critically ill children without prior model calibration using similar populations (26). In the study by Hughes et al., they evaluated the predictive performance of nine published pharmacokinetic models for vancomycin in a cohort of infants. Only two models showed an acceptable level of precision (27). Likewise, in the study by Hahn et al., they evaluated the predictive performance of two pharmacokinetic models in a cohort of pediatric patients; both models tested showed an acceptable level of bias and precision (28).

Several limitations should be acknowledged in this study. First, this was a single-center retrospective study, which may limit the generalizability of the findings to other settings. Second, the sample size for the a posteriori analyses was relatively small. Third, we only evaluated one dosing calculator; although other Bayesian dose calculators exist, they are not freely accessible and were therefore not included in this analysis. In addition, because this study used retrospective routine TDM records, factors such as acute illness severity, fluid resuscitation, or possible sampling or documentation errors were not consistently available. Prospective studies with detailed clinical documentation are warranted to better identify determinants of prediction accuracy. Finally, ARC was defined using the Schwartz equation based on serum creatinine, which may not always reflect true renal function in children. Emerging data suggest that cystatin C may provide a more accurate and dynamic marker of renal function in this population, and its use may further refine ARC diagnosis and dosing strategies (29). However, cystatin C measurement is not yet routinely implemented in clinical practice. Future studies should include prospective, multicenter studies to validate findings and improve generalizability. Studies evaluating different Bayesian dosing platforms in pediatric populations are also needed. Furthermore, integrating alternative biomarkers such as cystatin C into pharmacokinetic models could improve renal function assessment. Lastly, future investigations should assess the impact of Bayesian-guided dosing on outcomes such as target attainment, therapeutic efficacy, cost-effectiveness, and toxicity reduction.

In conclusion, the Bayesian dose calculator exhibited high bias in *a priori* predictions, often overestimating vancomycin concentrations, suggesting that it is not appropriate for determining initial dosing regimens. However, the incorporation of even a single measured drug concentration substantially improved the predictive accuracy across all subgroups. These findings support the use of the NextDose application with TDM to personalize vancomycin dose adjustments in pediatric patients, including those with ARC.

## Data Availability

The raw data supporting the conclusions of this article will be made available by the authors, without undue reservation.
